# Perspectives of adults aged 55+ on plant-based diets rich in protein

**DOI:** 10.1038/s41598-025-17087-x

**Published:** 2025-08-27

**Authors:** Sophie L. van Oppenraaij, Marije Putker, Anne van Schaik, Peter J. M. Weijs, Sjors Verlaan

**Affiliations:** 1 Department of Nutrition and Dietetics, Faculty of Health, Sport and Physical Activity, Amsterdam University of Applied Sciences, Dr. Meurerlaan 8, Amsterdam, 1067 SM The Netherlands; 2 Aging and Later Life, Amsterdam Public Health, Vrije Universiteit, Amsterdam, The Netherlands; 3 Department of Nutrition and Dietetics, Amsterdam University Medical Centers, Vrije Universiteit, Amsterdam, The Netherlands; 4 Department of Medical BioSciences, Radboud University Medical Center, Nijmegen, The Netherlands

**Keywords:** Plant-based diet, Protein intake, Healthy aging, Focus groups, Muscle preservation, Behaviour change, Nutrition, Psychology and behaviour

## Abstract

An increased protein intake is required during aging to preserve muscle mass. A predominantly plant-based diet is beneficial for the climate and may lower the risk of certain non-communicable diseases, but could also lead to protein below recommendations. This study seeks to elucidate facilitators and barriers in adults aged 55+ adopting a diet that is both predominantly plant-based and provides sufficient protein to preserve muscle mass. Three focus groups were conducted with 30 Dutch adults, aged 55–74 years. The focus groups explored participants’ views on protein-rich, (predominantly) plant-based diets. Participants discussed a range of topics, including perceptions of protein needs, attitudes toward plant-based foods, social influences, and practical considerations such as taste, cost, and habits. Three researchers independently thematically analysed the data and facilitators and barriers were mapped to the Capability (C), Opportunity (O), Motivation (M) – Behaviour (B) model. Health effects (M) and taste (M) were both the most dominant facilitators and barriers regarding transition towards a more plant-based, protein-rich diet. Knowledge (C) and social influences (O) were important factors that influence dietary choices regarding the other COM-B model components. Environmental benefits (M) and animal welfare (M) were mentioned as additional benefits rather than as main facilitator. Participants expressed a clear need for enhanced knowledge and tools about the health and environmental benefits of plant-based, protein-rich diets. Improving taste and highlighting health benefits are key to fostering positive attitudes and encouraging the adoption of protein-rich, plant-based diets among adults aged 55+.

## Introduction

Maintaining muscle mass, strength, and function is a prerequisite during aging to actively participate in society. Sarcopenia, defined by the age-related decline in muscle mass, strength, and function, is associated with higher chances of physical disability, falls, fractures, mortality, and with a lower quality of life^1^. Sufficient protein intake is essential to preserve and build muscle mass. Currently, 0.83 g protein per kg body weight per day (g/kg/d) is recommended for all adults^2–4^. To maintain and (re)gain muscle, however, older adults need more dietary protein than younger people – at least 1.0 g/kg/d, with higher intakes – up to 2.0 g/kg/d – recommended for those who are ill or physically active^5,6^. For older adults it is challenging to meet those recommendations^5–7^.

Adequate protein intake is usually realized by increasing intake of animal-based food products or supplements, because those products have a high protein content^8–11^. When older adults were asked to increase their protein intake to the recommended intake of 1.2 g/kg/d, animal protein intake increased substantially (+ 26.6 ± 2.6 g). In contrast, plant-based protein intake showed little change (+ 1.9 ± 1.1 g), resulting in a higher proportion of animal-based proteins in the diet^12^. Specifically, the proportion of protein derived from plant sources decreased from 36.6 to 28.6%. Accomplishing a similar increase with plant-based protein may be challenging, as protein content is higher in animal-based products compared to plant-based products^4,13–16^. Accordingly, research has shown that as diets become more plant-based, total protein intake often decreases^17,18^. Furthermore, the protein quality, digestibility, and absorption is also higher in animal-based products compared to plant-based products (i.e., soy, legumes, grains). However, animal-based food production contributes substantially to global greenhouse gas emissions^19,20^. Partly replacing animal-based food products with plant-based food, such as vegetables, legumes, and nuts, is a way to lower the environmental footprint and the risk of non-communicable diseases (i.e., cardiovascular diseases, type-2 diabetes, and cancer)^19–21^. To achieve the future climate goals, policy makers and health councils recommend to consume 60% plant-based proteins^20,22–24^. For older adults especially, achieving adequate protein intake remains essential while consuming ≥ 60% plant-based proteins^5,6^.

Previous studies, primarily among young adults, indicate that key motivations for reducing or eliminating animal-based products include animal welfare, health benefits, and environmental concerns^25,26^. A recent systematic review in adults aged 18–65 years further identified habits, uncertainty about food choices, and limited knowledge of nutritional intake and requirements as major barriers to adopting a plant-based diet^27^. Specifically, one focus group study found that health and appetite were the main reasons for reducing meat intake among adults aged 60+^28^, while a large survey showed that for adults aged 45+, knowledge, habits, and social influences were key barriers, with health being the primary perceived benefit of eating plant-based^29^. A qualitative research on increasing protein intake in older adults highlights dietary habits, awareness of health benefits, and product characteristics (taste, packaging, convenience) as key influencing factors^30^. To date, existing literature lacks insights on how older adults perceive a (predominantly) plant-based diet, defined as a dietary pattern ranging from meat-free diets to fully plant-based (vegan) diets^31^, while maintaining adequate protein intake. Additionally, while Dutch adults generally have a moderate protein intake, averaging around 1.0 g/kg/d, they obtain approximately 60% of their protein from animal sources^7^. A shift toward a more plant-based diet, as recommended by the Dutch Health Council and EAT Lancet Commission, may risk reducing overall protein intake to levels below the recommended intake for older adults. Consequently, this study seeks to elucidate facilitators and barriers in adults aged 55 + adopting a diet that is both predominantly plant-based and rich in protein.

## Methods

### Design

This qualitative study investigated the current perspectives of adults aged 55 + and to assess barriers and facilitators regarding predominantly plant-based diets rich in protein. We selected the age range starting from 55 years, as research indicates that muscle mass and strength can begin to decline from as early as age 30, with more pronounced losses typically occurring from around age 50 to 60^32,33^. Including adults from age 55 allows us to capture this critical period where muscle preservation becomes increasingly important. A qualitative design was chosen since this method is used to explore experiences, perceptions, and behaviour^34^. Focus groups were conducted since eliciting discussion is known to be effective in this method^35^. This study adheres to the Consolidated Criteria for Reporting Qualitative Research (COREQ) guidelines^36,37^. The study is in accordance with the ethical regulation and guidelines. Full ethical approval was granted by the HAS Green Academy (University of Applied Sciences) Ethics Committee (HEAC P2022 27).

### Recruitment

Individuals were included if they were 55 years or older and had sufficient proficiency in Dutch. Participants were recruited through university networks and a public fitness centre. The fitness centre was selected due to its high attendance of older adults, which allowed for the convenient organization of a focus group immediately following a fitness class. One of the researchers (SV) communicated with the fitness centre to distribute informational materials for recruitment purposes. These materials included an invitation to participate in a study on protein-rich and plant-based nutrition and enabled interested participants to either provide their contact information or contact the researcher directly to express their interest. Participants recruited via university networks received the same information. Purposive sampling was used to achieve gender balance, since women are more likely to eat more plant-based^38,39^.

### Data collection

Between April 2023 and June 2023, three focus groups were organized in the Netherlands with Dutch speaking individuals. An additional questionnaire was used to collect demographic characteristics, including age, gender, dietary habits (number of days eating meat per week), and physical activity (frequency of exercise per week). A semi-structured interview guide was developed by the research team to facilitate discussions. The questions in the interview guide are linked to the different components of the Capability, Opportunity, and Motivation – Behaviour (COM-B) model^40^. The interview guide was tested with two individuals separately and refined accordingly. The interview guide included topics regarding skills, experience, barriers and facilitators on protein-rich and (more) plant-based diets (Table 1). Participants were provided with an informational letter describing the study’s purpose and an informed consent was subsequently requested from each candidate participant. Audio recording devices were used to record all interactions in the focus groups after consent of each participant. The first focus group was conducted in person at the public fitness centre, while the second and third were held online using Microsoft Teams. A private room at the fitness centre was utilized for the session to ensure privacy and minimize background noise during the recordings. Focus groups typically include 5–8 participants, with a common range of 4–12^41^. We targeted the upper end of the group size range, as this fosters interaction and thus richer, deeper data by allowing participants to build on one another’s responses^41,42^. Sessions continued until data saturation was reached, with most themes typically emerging within three focus groups^43^. Despite the difference in format and location, the second session already showed substantial overlap with the themes identified in the first, and the third session largely confirmed these findings, with little new information emerging. To support engagement during the online sessions, we used several strategies, including ensuring that participants’ cameras and microphones were functioning and using active moderation to encourage participation^44^. Based on this consistency, we concluded that thematic saturation had been reached and did not organize additional focus groups.

**Table 1:** Discussion guide.

Main topic	Subtopic	Questions
Protein-rich diets	Skills and implementation	How do you interpret the term ‘protein-rich’? (C) What, in your opinion, are protein-rich products? (C) What do you think is the importance of consuming more proteins? Could there also be disadvantages? (C/M)
	Experience and considerations	Which protein-rich products are included in your daily diet? (C) Are you actively monitoring the amount of protein you consume daily? (C/M) Would you want to eat more protein, why or why not? (M) What role does your social environment play when you want to eat more protein? (O)
Plant-based and vegetarian diets	Skills and implementation	When you think of plant-based food, what comes to mind? What kind of products are these? (C) What are the health effects of a plant-based/vegetarian diet? Could there also be disadvantages? (C) Do you occasionally prepare a plant-based/vegetarian dinner? No: Do you think you could? What would you need? (C/O) Yes: How do you do this? What does the meal look like? (C/O) Imagine you could not eat animal products for breakfast and lunch, what would those meals then look like? (C)
	Experience and considerations	Do you consider the amount of animal-origin products when you buy groceries or eat a meal? (M) Which animal-origin products are included in your diet? Why do you choose to eat animal products? (M) How would you feel about using fewer animal products? (M) Would you want to eat more plant-based, why or why not? (M) What role does your environment play when you want to eat more plant-based protein? (O)
Plant-based and vegetarian diets rich in protein	Facilitators	When do you successfully adhere to a diet according to you? What factors contribute to this? (C) What would help you to consume more proteins/plant-based products? (C)
	Barriers	What factors might cause you to prematurely stop following a diet? (C/O/M) What are, in your opinion, disadvantages to a plant-based/vegetarian diet for you? What do you find off-putting? (M) Are there factors that could make following a more plant-based and vegetarian diet challenging? (C/O/M) Do you feel that you have enough knowledge to independently switch products, replacing animal proteins with plant-based proteins? If not, what information is missing? (C)
	Recommendations	Suppose you want to replace (some of) your meat products by plant-based and protein-rich alternatives. How would you go about finding (valid) information about an alternative product? (C) You are going to follow a vegetarian and protein-rich diet. Where do you start, and what do you need to follow this diet? (C) Imagine that you need to track your animal-based and plant-based protein intake. In what ways would you track this? (C)

*C* capability, *M* motivation, *O* opportunity.

### Data management

The audio recordings were erased 30 days after transcriptions. The analysis was carried out using anonymized transcripts. Anonymization was achieved replacing the participants names with a number. The facilitation of the focus groups was undertaken (moderated) by two researchers (SLVO and MP). One of the investigators (SLVO) attended specific courses on executing and analysing focus groups and the other researcher (MP) had experience with moderating focus groups in her previous work. The research team present at the focus groups included a PhD candidate (SLVO), two bachelor students in Nutrition & Dietetics (MP and AVS), and a senior researcher (SV). All researchers maintained neutral about the opinions of the study participants, who perceived them as researchers. The professions of the researchers were disclosed only after the focus groups to minimize bias in participants’ responses. The participants were solely informed that the researchers were seeking their perspectives and insights regarding predominantly plant-based diets rich in protein. Participants were informed that a (predominantly) plant-based diet refers to dietary patterns ranging from meat-free diets to fully plant-based (vegan) diets^31^. Participants were first invited to share their interpretations and experiences in response to open-ended questions from the discussion guide. This allowed spontaneous mention of protein-rich foods and plant-based products, including traditional sources and processed meat alternatives. If not raised, moderators used follow-up prompts to ensure these topics were addressed.

### Data analysis

The focus group discussions were analysed via thematic analysis, following a two-step coding approach^45^. Three researchers (MP, AVS, and SLVO) independently coded the data using an inductive approach to identify emerging patterns and themes. The coding process was conducted iteratively and facilitated using MaxQDA software (MaxQDA Analytics Pro 2022 Network). In the first phase, the researchers became familiar with the data by reviewing field notes taken during the focus groups. These notes were revisited before coding to support data familiarization. Then, inductive coding was applied, where initial codes were generated directly from the data without a predefined framework. In the second phase, the inductive codes were deductively categorized into the three components of the COM-B model: Capability, Opportunity, and Motivation^40^. Each code was further classified as either a facilitator or a barrier, depending on the context in which it appeared. Discrepancies in classification were discussed and resolved through consensus among the researchers. This stepwise approach ensured that participant-driven insights were first captured through open coding before being systematically mapped onto an established behavioural framework for interpretation. Code frequencies were recorded for transparency. All identified codes were included in the analysis.

## Results

A total of 30 individuals aged 55 years or older participated in the focus group discussions. This encompassed three focus groups, comprising 9, 10 and 11 participants, respectively. The duration of these focus group sessions were between 80 and 100 min. Two participants did not attend the sessions they had subscribed to, since they were not able to attend at the specified time anymore due to unknown personal reasons.

There were 14 male and 16 female participants in total, with a similar distribution over the focus groups. The median age of the participants was 63 years, with an interquartile range (IQR) of 60–67 years. All demographics are shown in Table 2. Participant recruitment predominantly occurred through university networks (*n* = 19), whereas the remaining participants (*n* = 11) were recruited through the public fitness centre.

**Table 2:** Demographic characteristics.

Demographic	Median (IQR) or n (%)
Gender	
Male	14 (47%)
Female	16 (53%)
Age (years)	
Median (IQR)	63 (60–67)
55–59	7 (23%)
60–69	17 (57%)
70–79	6 (30%)
Times of exercising per week	
1	2 (7%)
2–3	19 (63%)
4–6	9 (30%)
≥ 7	0 (0%)
Days of eating meat per week	
< 1	7 (23%)
1–3	8 (27%)
4–6	13 (43%)
7	2 (7%)

*IQR* interquartile range.

**Fig. 1:**
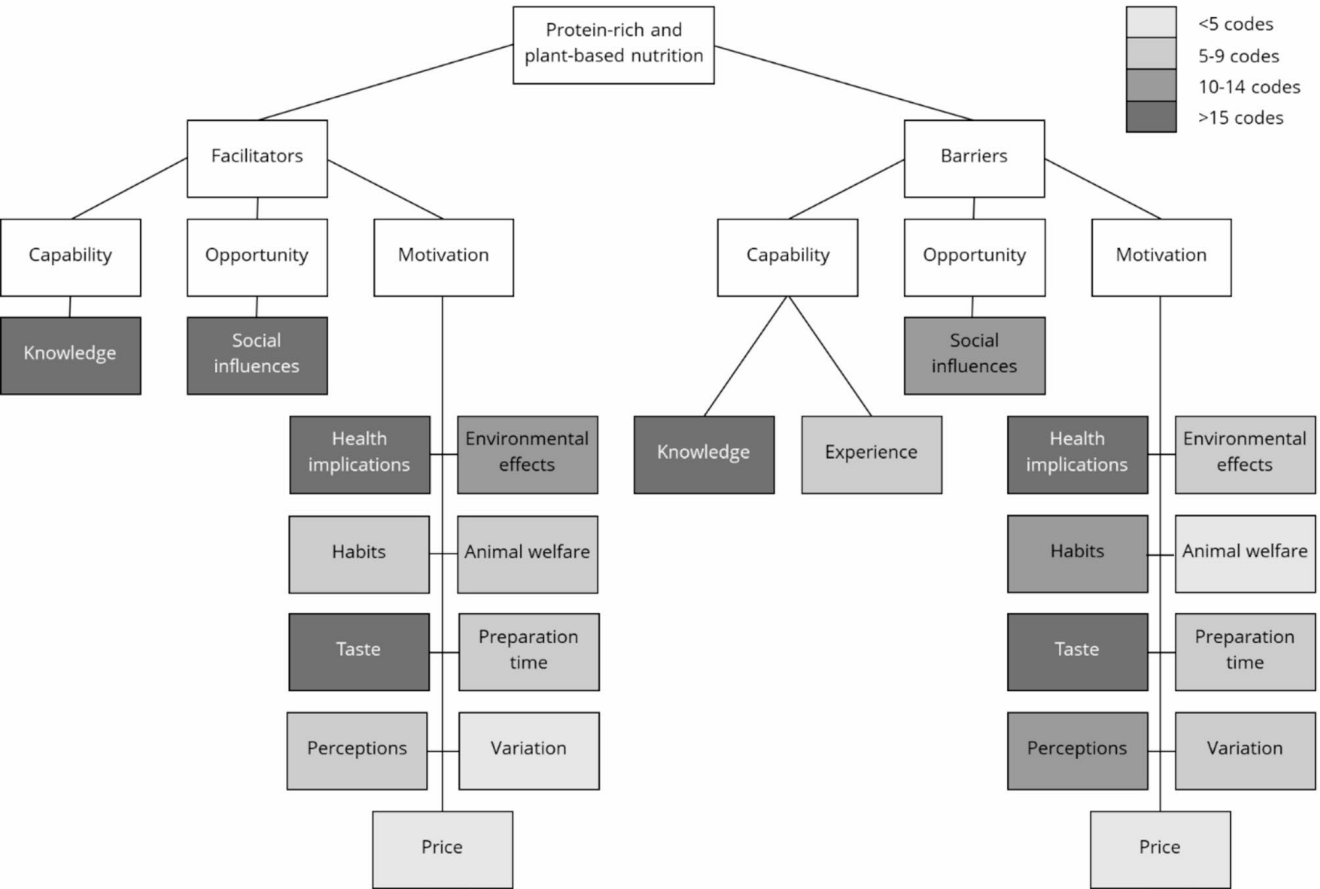
Themes and subthemes. Frequencies are the number of unique participants who expressed a code at least once across all sessions.

The focus groups with adults aged 55 + provided insight into the facilitators and barriers regarding predominantly plant-based diets rich in protein. The major themes that arose from the data are shown in Fig. 1.

### Facilitators

#### Capability

##### Knowledge

Participants indicated that possessing knowledge about a healthy diet, including food products and (plant-based) protein sources, significantly facilitated their choices towards plant-based and protein-rich foods. During the focus group discussions, it was evident that decisions about dietary selections were influenced by an understanding of the health benefits and drawbacks of various food products. Familiarity with dietary guidelines, such as the Dutch ‘Wheel of Five’, and the use of digital tools for diet tracking substantially helped participants in making more conscious dietary choices.

**Table:** 

***R6 (M, 64y, 4-6d meat/week):*** * "When you look at the ‘Wheel of Five’ [Dutch Dietary Guidelines], you see that you need to eat a handful of nuts daily and that you should eat legumes once a week because of their tremendous richness in proteins and vitamins."*	

**Table:** 

***R30 (M, 59y, 4-6d meat/week):*** * “It’s also important for maintaining your muscles, so if you eat too little protein, then you also lose muscle.”*	

Moreover, a strong understanding of the nutritional value of food products encouraged participants to choose for plant-based and protein-rich choices. The ability to identify protein-rich foods, such as dairy products, legumes, and meat substitutes, helped them in making these choices.

**Table:** 

***R15 (F, 57y, 4-6d meat/week):*** * “I often eat vegetarian alternatives. What I sometimes check is the amount of proteins in the product, and usually, it’s very little. I think it should be at least 15%. There aren’t many like that.”*	

#### Opportunity

##### Social influences

The influence of social environments, such as family and close contacts, played a crucial role in dietary decisions. Participants reported significant changes in their eating habits due to the influence of partners or family members encouraging to reduce consumption of animal-based food products. Furthermore, external sources of information, such as television, played a role in shaping dietary decisions by influencing awareness of the health effects of protein. Additionally, it was observed that social interactions were reciprocal, meaning that participants were both influenced by others (R17) and influenced others themselves (R21).

**Table:** 

***R17 (F, 60y, 1-3d meat/week):*** * "We started eating less meat because my youngest son refused meat, he suddenly said: ‘I don’t want to eat meat anymore.”*	

**Table:** 

***R21 (M, 63y, 1-3d meat/week):*** * "When I eat at someone’s place and they serve meat every week, I sometimes say: 'Well, I sometimes replace it with this [meat replacer], and that’s also quite tasty.' Often, they’ll give it a try too. I eat at my brother’s every week, and there I try to kindle that interest a bit each time."*	

#### Motivation

##### Health implications

During the focus groups, it became clear that health was for almost all participants one of the most important facilitator for making dietary choices. There was a strong consensus across all groups that health is the main driver in these choices. The participants that were trying to increase their protein intake were all motivated by the health effects, mainly to support muscle maintenance. Health effects were also a driver regarding eating more plant-based. However, not all participants connected plant-based eating to positive health effects.

**Table:** 

***R7 (M, 60y, 4-6d meat/week):*** * “Nowadays, they [our generation] are much more aware of the animal products and the plant-based products that are healthier.”*	

##### Environmental effects and animal welfare

Another motivation among participants for adopting a plant-based diet was the desire to minimize environmental impact and promote animal welfare. A few participants cited these intrinsic factors as one of the primary reasons for reducing the consumption of animal-based products. The majority agreed to this additional benefit, but this was mostly not their main driver.

**Table:** 

***R17 (F, 60, 1-3d meat/week):*** * "There are quite a few reasons to eat more plant-based foods, particularly because of the climate and the impact of livestock farming. For the sake of the climate, and future generations, you simply have to reduce consumption of animal products, especially those that contribute significantly to emissions, and particularly the leaner products. So anything that comes from cows, or sheep, or goats, those are the products you should cut back on the most."*	

**Table:** 

***R24 (M, 62y <1d meat/week):*** * "I consider it important that a product is not animal-based, meaning that no animals were slaughtered, and thus there is no associated emissions. Additionally, it is significant to me that the product is produced responsibly and that its environmental impact is minimal. Manufacturers should provide evidence that they adhere strictly to these."*	

##### Taste, habits, price, Preparation time

Taste, habits, price, and preparation time were not seen as barriers among some participants, and could even be seen as facilitating factors. Some participants expressed a preference for the taste of both animal-based products, such as meat and dairy, and plant-based products, such as meat substitutes. Furthermore, certain participants had modified their habits without much effort. For instance, one individual adopted a vegetarian diet due to the influence of someone he lived with and this led to eating no more meat. For some participants, neither price nor preparation time posed concerns. Participants were also willing to spend more on plant-based foods, since they were also willing to spend more on organic foods. Although some noted that preparing predominantly plant-based dishes, e.g., with multiple vegetables, might require more time, those with adequate time did not view this as a barrier.

**Table:** 

***R9 (M, 66y, <1d meat/week):*** * "My previous girlfriend was vegetarian, so naturally, meat no longer appeared on the table. And after living with someone for 25 years, you eventually move away from meat. And now, I actually don’t want it anymore."*	

### Barriers

#### Capability

##### Knowledge

A lack of accurate knowledge or misunderstandings about plant-based and protein-rich diets sometimes led to reduced intake of protein and plant-based food products. Misconceptions about the healthfulness of plant-based diets were evident.

**Table:** 

***I:*** * "Suppose you were to eat more plant-based foods, how would you go about it?"*	
***R16 (F, 67y, 4-6d meat/week):*** * "I would first need to see what exactly plant-based entails.”*	

**Table:** 

***R10 (M, 73y, 4-6d meat/week):*** * “I sometimes see people who don’t eat any meat at all, and then I think to myself: what color is your face? Gray, dull. There are so many young people who look utterly pallid. They are not healthy, not fit, like we used to be.”*	

##### Experience

Additionally, a notable barrier was the lack of experience with plant-based cooking. Participants with no prior experience in preparing meat substitutes expressed reluctance, despite a willingness to explore these culinary practices.

**Table:** 

***R5 (F, 63y, 4-6d meat/week): *** *“ No, I wouldn’t see that [plant-based cooking] as a barrier. However, I wouldn’t know what to use right now. I really have no idea how to replace that turkey.”*	

**Table:** 

***R11 (M, 74y, 4-6d meat/week):*** * "I have never eaten a meat substitute, as it is designated, no. Actually, I find it so unappetizing to look at. I'd rather not. I would really have to delve into how to do that.”*	

#### Opportunity

##### Social influences

The influence of social environments, such as family and close contacts, was also seen as a barrier regarding dietary decisions. Social pressure and autonomy in dietary decisions also posed challenges. Some participants felt constrained by their family roles or societal expectations, impacting their willingness to adopt plant-based diets. Moreover, social influences could also work the other way around, where someone played an influential role in dietary choices among others. For example, one participant always cooks and would never use a meat substitute, influencing the dietary choices of others.

**Table:** 

***R2 (F, 57y, 1-3d meat/week):*** * "I have no say in our diet. My wife does the cooking, and she has her own ideas about it."*	

**Table:** 

***R20 (M, 73, 4-6d meat/week):*** * "If it gets to the point where it’s like 'you have to eat plant-based!', then I think, well no thanks. Like: I'll decide for myself. Who are you to dictate to me, those kinds of thoughts."*	

#### Motivation

##### Health implications

As noted before, almost all participants made dietary decisions based on the health effects. It was therefore noted that participants would only change their dietary behaviour towards a more plant-based diet if this has positive effects on their health. If there is a chance that it is not healthier, most would not bother to change. Besides doubts that it is healthier, there were also concerns about the health drawbacks of processed plant-based foods, for example meat alternatives. Issues such as high salt, sugar, and saturated fat content were experiences as barriers by the participants. Additionally, the lower protein content in plant-based products was mentioned as barrier.

**Table:** 

***R6 (M, 64y, 4-6d meat/week):*** * “Well, I just went to my fridge to check out all the plant-based alternatives. And, I must say I never read the labels very carefully, but here I have three products made from coconut oil. They contain an incredible amount of saturated fat. (…) So, if you want to eat everything plant-based, you’re also faced with other problems.”*	

**Table:** 

***R15 (F, 57y, 4-6d meat/week):*** * “I have the impression that if you want to consume enough proteins, you need to eat such enormous amounts of vegetables and grains to reach the necessary levels of proteins that you need.”*	

##### Taste preferences

The taste of food emerged as a paramount factor influencing dietary choices. Nearly all participants cited taste as a key factor when making decisions regarding their diet. There was a strong consensus in every group that taste influences these dietary choices. Some participants expressed a preference for the taste of meat and were less inclined to consider plant-based alternatives unless they matched their flavour expectations. The opinion based on experience or perception of meat alternatives was rather negative. Moreover, not all participants expressed concerns regarding protein content when replacing protein-rich products with plant-based alternatives.

**Table:** 

***R19 (M, 63y, 7d meat/week):*** * “Well, I think what’s very important to me is what the taste would be. Yes, I think that’s very important to me. I'm not really into healthy eating. I just don’t like the taste. If I were to eat plant-based at all.”*	
***I:*** * “And what would be your consideration for deciding whether or not to do that?”*	
***R19 (M, 63y, 7d meat/week):*** * “Yes, definitely health then. That is indeed the most important thing for me, yes.”*	

**Table:** 

***R18 (M, 63y, 4-6d meat/week):*** * “But for me, it’s not necessary at all, always having a piece of meat. That mushroom burger may not be very high in protein, but that’s not what I’m concerned about. It’s mainly about the taste for me.”*	

##### Perceptions

Several participants had negative perceptions towards predominantly plant-based diets. The notion that plant-based foods were less appetizing or inferior to meat-based options was a significant barrier. Despite these reservations, there were concurrent motivations to adopt dietary changes.

**Table:** 

***R17 (F, 60y, 1-3d meat/week):*** * “When I look at the shelf with those meat substitutes, I think: well… to me, that doesn’t look healthy. All with those crispy coatings. And then I think: well, all the stuff around it. What is actually in it? Very processed!”*	

**Table:** 

***R3 (M, 55y, 7d meat/week):*** * "When you coldly call it 'plant-based food’… It has an emotional value of zero for me. It’s just not appealing. The footprint idea, sure, fine, but fun is something else."*	

**Table:** 

***R7 (M, 60y, 4-6d meat/week):*** * "I believe that eating behaviour is deeply ingrained in people. And so, you need to try and gently coax them out of it. If you make a drastic move, then… When I hear you talk about a fully plant-based diet, well, that makes my hackles rise. (…) But if you say, I want you to try doing it fifty-fifty, that sounds a lot better."*	

##### Dietary habits

The reluctance to adopt a (more) plant-based diet was further reinforced by dietary habits. This was also seen for increasing protein intake.

**Table:** 

***R7 (M, 60y, 4-6d meat/week):*** * "I do cook a lot, but there’s always some kind of meat or fish in it. But I don’t know. It’s just a habit. I don’t really think about cooking vegetarian. It seems too complicated to me, I guess."*	

##### Environmental effects

Regarding the motivation to reducing animal-based products for environmental benefits, there was a noted disparity between participants’ perceptions and current scientific knowledge about these environmental benefits, which sometimes led to scepticism about their efficacy. Additionally, health was often noted as more important than environmental benefits.

**Table:** 

***R3 (M, 55y, 7d meat/week):*** * “I really find it a turn-off when you eventually realize that a product is also terribly bad for the environment. That’s a real deal-breaker for me and a reason to skip the product next time.”*	

**Table:** 

***R2 (F, 57y, 1-3d meat/week):*** * “Look, if the environmental footprint becomes smaller, but it’s unhealthier, well, then I wouldn’t really consider switching to a plant based diet.”*	

##### Preparation time and variation

Some participants perceived that plant-based nutrition required longer preparation times, which they identified as a barrier. Additionally, a lack of variety in plant-based options when eating out was seen as another deterrent by other participants.

**Table:** 

***R23 (M, 60y, <1d meat/week):*** * “I'm going somewhere and then we’ll have lunch somewhere. Yes, then sometimes making choices can be very difficult when you’re out.”*	

#### Recommendations

During the focus groups, opinions varied on possible methods for transitioning to a protein-rich and more plant-based diet. Although approaches varied, it was clear that most participants required information on how to integrate more plant-based, protein-rich foods into their diets. To facilitate this, participants expressed a preference for both paper-based and digital tools.

##### Recipes, meal examples, and product lists

Some participants expressed a desire for a range of meal examples and recipes. There was also interest in lists of food products with protein-rich and plant-based options. Additionally, participants highlighted the need for a ‘replacement list’ that could guide the substitution of animal-based products with equivalent alternatives in terms of protein quantity. It was also apparent that participants valued having multiple options available.

**Table:** 

***R20 (M, 73, 4-6d meat/week):*** * “Just have a healthy diet put together by a dietitian. And especially make the meals available.”*	

**Table:** 

***R28 (F, 60y, 1-3d meat/week):*** * “Yes, you know, if I, hypothetically speaking, had a list with, well, mainly what and when is enough, right? So if you were to stop eating cheese. How many nuts would you need to eat or something? I'm just saying something random, but yeah, that.”*	

##### Diet tracking and general knowledge

Some participants preferred to monitor their diet to enhance their nutritional knowledge. Enhancing knowledge was a recurring theme, with participants favouring different methods.

**Table:** 

***R1 (M, 60y, 1-3d meat/week):*** * “Having a set diet often means you’re not very aware of what you’re eating. I find it nicer to be more aware. (…) A digital diary really makes you aware of what you’re eating. You enter something and then you see how many calories or how much sugar it contains. Then you think oh wait. I now consciously choose a different variation. (…) It makes you more aware of what you’re eating.”*	

**Table:** 

***R22 (F, 63y, 4-6d meat/week):*** * “I mean, I think if you start doing recipes at some point, you’ve tried everything once and then you drop out, whereas if you just have more general knowledge and you can apply that to what you like and what combines well, it’s easier to maintain.”*	

##### Product information

Participants also indicated a need for transparent information about food products to simplify making decisions.

**Table:** 

***R3 (M, 55y, 7d meat/week):*** * “I would strongly support having products display their environmental impact alongside the Nutri-Score. And also: what did this product cost? That should be fairly simple to do. But often, supermarkets and manufacturers play a concealing tactic with this.”*	

## Discussion

In exploring the views of adults aged 55 + on transitioning towards more plant-based diets rich in protein, multiple facilitators and barriers emerged. The focus groups showed a strong consensus that health was the most important driver to increase protein intake and change towards a more plant-based diet. If altering their diet would not improve their health, most participants would not bother to change it. It was observed that taste was the other most important driver for making dietary decisions, which could be seen as a barrier regarding transitioning towards a more plant-based diet rich in protein. Other factors that influence dietary choices were knowledge, social influences, and dietary habits. Environmental benefits and animal welfare were mentioned as additional benefit rather than as main driver. When asked for recommendations, participants expressed a clear need for enhanced knowledge and diverse types of tools, which could contribute to independently increasing protein-rich and plant-based dietary products.

Using the COM-B model to synthesize the findings, it becomes evident that both facilitators and barriers are present at all three components. Most factors clustered under motivation. Nevertheless, knowledge and social influences within the capability and opportunity categories emerged as critical factors in this study.

### Capability

The focus groups showed that sufficient understanding of the nutritional value of foods (i.e., basic level of knowledge regarding healthy eating, plant-based diets, and common dietary sources of protein) significantly supports the likelihood of adopting a more plant-based diet rich in protein. This is in line with existing literature that suggests that food product knowledge plays an important role in influencing consumer behaviour^46,47^. A higher level of product knowledge positively correlates with purchasing decisions. Awareness of the health benefits associated with plant-based diets rich in protein has also been shown to influence dietary choices in our study. This aligns with previous research indicating that health benefits are the primary advantages associated with plant-based diets^29^. Additionally, research indicates that providing information about the health effects of protein resulted in increased protein consumption^48,49^. Conversely, according to the focus groups, insufficient or inaccurate knowledge about plant-based or protein-rich diets occasionally resulted in a lower intake of these foods. Lower protein intake when there is no or inaccurate knowledge about this topic is also mentioned in other studies^50–52^. Moreover, a systematic review showed that multiple studies identified lack of knowledge as a barrier to eat more plant-based^27^.

### Opportunity

Having and/or being a role model is seen as another important promoting factor to eat more plant-based and protein-rich food products. If someone in the participant’s environment consumes more plant-based foods, or specifically asks for more plant-based foods, the participant’s willingness to follow appears to be higher. Additionally, awareness of the importance of proteins, provided by media, health professionals, and/or social contact, was noted to contribute to a higher protein intake among participants in our focus groups. These results are supported by a review on the effect of a role model on food choices, which concludes that a large part of someone’s food intake is determined by social influences^53^. Other research confirms that having a social network around someone with individuals who eat less or no meat is a strong predictor of that person’s reduction in meat consumption^54^. This research^54^ also described that less meat was consumed if someone had a vegetarian friend in their environment, again confirming the effect of a role model. The focus groups, however, showed that social influences can also be a barrier to change a diet. Being pushed into a plant-based diet or relying on others to cook was seen as a difficulty when changing a diet. This aligns with evidence showing that when household members are unable or unwilling to modify their diets, it becomes difficult for someone to change their eating pattern^29^.

### Motivation

Health effects were for almost all participants the most important factor to make dietary choices. This study showed that health benefits were a facilitator to eat more protein and/or plant-based foods, which is confirmed by other studies^28–30^. However, in contrast to proteins, where most people believe in the positive health effects of them, plant-based diets are not always perceived healthy. Different opinions were shown on this topic. Some participants had the opinion that excluding meat from a diet would be unhealthy, whereas others were concerned about the protein content of more plant-based diets or the health drawbacks of meat replacers. Concerns about the lower nutritional quality of plant-based diets is also seen in other research^29^. Despite the health effects of a diet with a larger proportion of plant-based food products because of the higher fibre, unsaturated fat, folate, vitamin C and E, and magnesium intake, it is also shown that (more) plant-based diets contain lower amounts of protein, iron, eicosapentaenoic acid (EPA), docosahexaenoic acid (DHA), vitamin B_12_, calcium and iodine^55^.

Additionally, taste was for most participants important for making dietary decisions. For some participants it was the most important factor and were not concerned about the health effects of food. Taste as motive to eat animal-based products is also confirmed by other studies^29,56^. Habits also emerged as an important barrier. Participants appeared comfortable with their routines and habits, which complicates the transition towards a more plant-based and protein-rich diet. This outcome is in line with previous research on barriers for transitioning towards a plant-based diet, which found one of the main barriers to consuming plant-based food is the reluctance to change eating habits or routines^29^. This study revealed that resistance to changing existing habits was the primary barrier identified by respondents aged 60–91 years, and the third most common among those aged 45–59, comparable to the age in our study.

This study identified negative perceptions participants have with plant-based foods, particularly meat substitutes, which they perceive as artificial, overly processed, unappetizing, and synthetic. These views are supported by previous research which confirms that meat substitutes are generally viewed more negatively than meat^57^. Additionally, the preference for whole foods over processed foods was also reflected in a study examining perceptions of processed foods^58^. When discussing their impressions of meat substitutes, participants used terms such as ‘disgusting’, confirming the negative perceptions observed in this study. This aligns with aversion or the absence of stimulus, meaning that participants feel a certain degree of reluctance towards a more plant-based diet. This lack of motivation to reduce or eliminate meat consumption aligns with findings from prior research, which identified the enjoyment derived from eating meat and the challenges associated with reducing meat consumption as primary barriers^59^. Furthermore, another study highlights three principal barriers to reducing meat consumption: the belief that humans are naturally meat-eaters, the perception that plant-based foods lack flavour, and the pleasurable experience associated with consuming meat^60^. These findings highlight that both reluctance and the absence of stimulus significantly hinder the transition to more plant-based diets.

Previous research confirms that awareness of the climate impact of meat increased the likelihood of reducing meat consumption^54^. Additionally, two studies showed positive environmental impact was one of the three main motives to reduce these products^25,26^. The focus groups in this study showed that some participants were concerned about the impact of food on the environment but it was for most participant not their main driver in making dietary decisions. This is also seen in other research, where there is scepticism about the environmental impact^61,62^. One of these studies^61^ also showed that some participants did not believe in or were unaware of climate change. Some people in our focus group were not concerned with the impact of food on the climate, but no one expressed their doubts about scientific evidence of climate change.

Animal welfare emerged as a factor influencing only a few participants to reduce their intake of animal-based products, which is not in line with other studies where it was the most mentioned motive^25,26^. While no participants questioned this viewpoint, others maintained that consuming animal-based products is normal and justified by tradition. This perspective aligns with another study that argued that the consumption of animal-based foods is often rationalized as natural, normal, necessary, and nice^56^. The term natural refers to, among other things, the inherent nature of meat consumption, mirroring the opinions of some participants in our study.

### Recommendations

Enhancing knowledge appears crucial for enabling individuals to independently make plant-based and protein-rich dietary changes. Previous research confirms that a lack of information and knowledge about plant-based food is the most significant barrier to choosing such a diet^29^. Some participants want recipes, meal examples, and/or list with food products to choose from, while others prefer tracking their diet or learning more about a more plant-based diet rich in protein to be able to change their diet independently, which shows that one approach for all is not possible. However, there is to our knowledge insufficient research on the effectiveness of different types of tools (e.g. recipes, food records) on eating behaviour (e.g. increasing (plant-based) protein), whereby no comparison can be made. Participants also expressed the need for transparent information on food products about the environmental footprint and costs to simplify making decisions. The Dutch Health Council recently published a report in which it recommends implementing policy measures to achieve the current policy goal of a 50% plant-based and subsequent progress to 60% plant based, by e.g. adhering to a vegetarian diet including fish consumption once per week^24^. One policy measure to accelerate the transition could be the promotion of transparent information regarding the environmental impact of food products in conjunction with information on the nutritional product composition, including protein content. This would help consumers to become aware of the effects of their choices and encourage them to substitute products, thereby improving their environmental footprint, while having an adequate intake of protein and other nutrients.

### Strengths

Previous studies focused on either facilitators and barriers of increased protein intake^30^ or increased intake of plant-based foods^25–27^. Furthermore, studies on more plant-based foods did not focus on adults aged 55 + specifically. An important strength of this study was that, to our knowledge, this focus group study focused on the combination of two topics: plant-based nutrition and protein consumption and was focussed on people aged 55 + solely. The focus group approach allowed for an in-depth exploration of the perspectives of adults aged 55 + on adopting a predominantly plant-based diet while ensuring adequate protein intake.

### Limitations

During the interviews, discussions shifted between the participants’ protein consumption habits and their views on a more plant-based diet. Therefore, it was not always possible to address the connection between the topics. Additionally, the study participants may not fully represent the broader Dutch adult population aged 55+. In this study, 93% of the participants exercised twice or more per week, whereas 50% of the Dutch adults aged 50 years or older exercise weekly^39^. This suggests that the participants in this study might prioritize health more than the general Dutch population aged 55+, and making them more receptive to the protein transition. Another limitation of this study is the variation in focus group format, with one conducted in person and two conducted online. Differences in group dynamics and participant engagement between in-person and online settings may have influenced the depth or nature of the discussions. However, consistent themes emerged across all sessions, suggesting that this impact was limited.

### Future research

To better understand how the views expressed in this study translate into dietary behaviour, future research should focus on actual behaviour in real-life settings. Longitudinal or intervention studies could help clarify whether and how knowledge and attitudes toward plant-based and protein-rich diets lead to meaningful dietary changes over time.

## Conclusions

This qualitative study in adults aged 55 + elucidates that health effects and taste were both the most important facilitators and barriers to transition towards a more plant-based, protein-rich diet. Other factors that influence dietary choices and were more explicitly mentioned were knowledge, social influences, and dietary habits. Environmental benefits and animal welfare were mentioned as additional benefits rather than as main driver. This study largely confirms findings from previous research on the transition toward either more plant-based eating or increased protein intake, but uniquely combines both dietary shifts into a single transition goal. Improving the taste and highlighting health benefits of plant-based products are essential for fostering more positive attitudes and increasing the willingness to explore a wider range of plant-based foods rich in protein. Future research could explore strategies to improve the acceptability and implementation of protein-rich, plant-based diets and evaluate their effectiveness in this population.

Moreover, a strong understanding of the nutritional value of food products encouraged participants to choose for plant-based and protein-rich choices. The ability to identify protein-rich foods, such as dairy products, legumes, and meat substitutes, helped them in making these choices.

## Data Availability

The datasets generated and/or analysed during the current study are not publicly available due to participants not providing consent for data sharing but are available from the corresponding author upon reasonable request.
